# A phubbing scale tested in Bangladesh, Iran, and Pakistan: confirmatory factor, network, and Rasch analyses

**DOI:** 10.1186/s12888-023-05251-4

**Published:** 2023-10-18

**Authors:** Chung-Ying Lin, Mohammed A. Mamun, Firoj al Mamun, Irfan Ullah, Ismail Hosen, Najma Iqbal Malik, Abiha Fatima, Ali Poorebrahim, Morteza Pourgholami, Marc N Potenza, Amir H Pakpour

**Affiliations:** 1 Institute of Allied Health Sciences, College of Medicine, National Cheng Kung University, Tainan, 701 Taiwan; 2 Biostatistics Consulting Center, National Cheng Kung University Hospital, College of Medicine, National Cheng Kung University, Tainan, 701 Taiwan; 3 Department of Occupational Therapy, College of Medicine, National Cheng Kung University, Tainan, 701 Taiwan; 4 Department of Public Health, College of Medicine, National Cheng Kung University, Tainan, 701 Taiwan; 5 Department of Public Health and Informatics, CHINTA Research Bangladesh, Jahangirnagar University, Savar, Dhaka, Savar, Dhaka, Bangladesh; 6 Kabir Medical College, Gandhara University, Peshawar, Pakistan; 7 Department of Psychology, University of Sargodha, Sargodha, Pakistan; 8 Karachi Medical and Dental College, Karachi, Pakistan; 9 Guilan University of Medical Sciences, Rasht, Islamic Republic of Iran; 10 Department of Psychiatry, Yale School of Medicine, New Haven, CT USA; 11 Social Determinants of Health Research Center, Research Institute for Prevention of Non- Communicable Diseases, Qazvin University of Medical Sciences, Qazvin, Iran; 12 Department of Nursing, School of Health and Welfare, Jönköping University, Barnarpsgatan 39, Jönköping, 55111 Sweden

**Keywords:** Smartphone behaviors, Confirmatory factor analysis, Network analysis, Rasch model, Measurement invariance, Differential item functioning

## Abstract

**Background:**

Phubbing, a phenomenon of ignoring others in face-to-face conversations due to mobile phone use, can be assessed using a Phubbing Scale (PS). Recently, the PS has been shortened into an eight-item version, the PS-8. However, psychometric properties of the PS-8 among Iranian, Bangladeshi and Pakistani individuals remain understudied, especially using advanced psychometric testing, such as Rasch and network analyses.

**Methods:**

Participants residing in Iran, Bangladesh, and Pakistan (n = 1902; 50.4% females; mean age = 26.3 years) completed the PS-8 and the Internet Disorder Scale-Short Form (IDS9-SF) via an online survey. Network analysis was used to examine if PS-8 items were differentiated from IDS9-SF items; confirmatory factor analysis (CFA) was used to examine the factor structure and measurement invariance of the PS-8; Rasch modeling was used to examine the dimensionality of the PS-8 and differential item functioning (DIF).

**Results:**

Network analysis showed that PS-8 items were clustered together with a distance to the IDS9-SF items. The CFA results supported a two-factor structure of the PS-8, and the two-factor structure was found to be invariant across countries and women and men. Rasch model results indicated that the two PS-8 subscales were both unidimensional and did not display DIF across countries and gender/sex.

**Conclusion:**

The PS-8 is a feasible and robust instrument for healthcare providers, especially mental health professionals, to quickly assess and evaluate individuals’ phubbing behaviors.

**Supplementary Information:**

The online version contains supplementary material available at 10.1186/s12888-023-05251-4.

## Introduction

Technological advances have promoted use of smartphones and mobile phones (hereafter smartphone use also indicates mobile phone use unless explicitly mentioned) as smartphones offer entertainment, convenience and social rewards [1]. Surveys from BankMyCell et al [2] suggest that over 80% of individuals worldwide (> 6.6 billion) own at least one smartphone (not counting mobile phones) and over 90% (> 7.2 billion) own at least one mobile phone or smartphone. Smartphone use increased during the COVID-19 pandemic [3, 4], including with respect to remote meetings, online learning, and telehealth [5, 6]. As such, issues related to smartphone use (e.g., smartphone addiction or problematic use of smartphones) warrant consideration [7, 8], particularly with respect to mental health concerns.

Among concerns associated with smartphone use [9–13], phubbing may need additional attention from healthcare professionals. Phubbing, a merged word combining “phone” and “snubbing” [14, 15], has been defined as “ignoring other individuals by using a mobile phone during a face-to-face conversation” [16]. According to this definition, individuals who engage repeatedly in phubbing may experience impaired in-person social functioning. In other words, phubbing may reduce human contacts and promote problems in human interactions. Such impaired in-person interactions may promote mental health problems, and mental health professionals should consider impacts of phubbing.

Karadağ et al. [15] developed a Phubbing Scale (PS). Of the 10 PS items, two factors (with five items in each factor) were identified by principal component analysis [16]. PS scores with measures related to smartphone addiction, such as social media, internet, and gaming addictions [17–22]. Although the PS has been used across different countries to assess associations with addictions, relatively few studies have examined its psychometric properties [16].

To the best of the authors’ knowledge, the PS has been tested for its psychometric properties in a Spanish sample [19] and a cross-country sample with 20 countries involvement [23]. The two-factor structure of PS was confirmed in the Spanish sample [19]; however, this two-factor structure was not invariant across the 20-country sample [23]. Nevertheless, Błachnio et al. [23] found that a shortened PS (i.e., 8-item PS, abbreviated as PS-8) was invariant across the 20 countries. García-Castro et al. [16] confirmed that the PS-8 is a feasible instrument with good validity.

Among the psychometric testing methods used for examining the factor structure of the PS-8 (i.e., whether it is a one-factor or a two-factor structure), only confirmatory factor analysis (CFA) has been used. Therefore, although the PS-8 has been validated across 20 countries, empirical psychometric studies of the PS-8 remain limited. Specifically, two other advanced forms of psychometric testing (i.e., network [24–26] and Rasch analyses [27–31]) could be used to investigate further the factor structure of the PS-8 and to test that phubbing assessed by the PS-8 is different from internet addiction. Because a good instrument should have convergent evidence regarding its psychometric properties [32], it is important to have different psychometric methods support an instrument’s factor structure.

Apart from the lack of network and Rasch analyses for the PS-8, the present authors are not aware of any psychometric examinations of the PS-8 among Iranian and Bangladeshi individuals. In order to assist researchers and healthcare providers residing in Iran and Bangladesh to assess and evaluate phubbing, the PS-8 should be examined for these countries’ residents. Therefore, the present study aimed to examine the factor structure of the PS-8 using different psychometric testing methods (including CFA, network analysis, and Rasch analysis) across three countries (i.e., Bangladesh, Iran, and Pakistan). Additionally, the present study examined if the PS-8 has an invariant factor structure across the three countries. Furthermore, given sex-/gender-related differences in internet use (including socially) and the importance of considering sex-/gender-related effects, we explored DIF across women and men. We hypothesized the PS-8 would show a two-factor structure with invariance across different countries. Given prior findings indicating that the PS-8 was invariant across men and women in a Portuguese sample [16], we hypothesized invariance across men and women in the present study.

## Methods

### Participants and procedure

Study participants were adults from three countries (Iran, Bangladesh, and Pakistan). We compared the three countries because the three countries all locate in Southern part of Asian and share the same beliefs of Muslim. In this regard, their behaviors and psychological states are associated with their beliefs and may share similar psychological features and online behaviors. An online platform was used to collect data from March 2020 to December 2020. The study procedure has been reported elsewhere [26, 33]. All participants provided informed consent before participating in the study. The studies involving human participants were reviewed and approved by the Institute of Allergy and Clinical Immunology of Bangladesh, Department of Psychology, University of Sargodha, Sargodha, Pakistan, and Qazvin University of Medical Sciences.

### Measures: 8-item Phubbing Scale (PS-8)

The PS-8 contains 8 items assessed on a five-point Likert scale, of which scoring 1 indicates never and 5 indicates always. The PS-8 has been proposed to have a two-factor structure, with the first four items within a domain of Communication Disturbance and the last four times within a domain of Phone Obsession. Higher PS-8 scores reflect more severe phubbing. The PS-8 has been recently validated with a two-factor structure supported and satisfactory internal consistency (ω = 0.85 for Communication Disturbance and 0.76 for Phone Obsession) [16]. The 10-item version of the Phubbing Scale (i.e., PS) has been validated in the Iranian Persian [34] and Pakistan Urdu [23] languages.

### Internet Disorder Scale–short form (IDS9-SF)

The IDS9-SF contains 9 items assessed on a five-point Likert scale, of which scoring 1 indicates never and 5 indicates very often [35]. The IDS9-SF has been proposed to have a one-factor structure with all nine items loading on the same domain of internet addiction. A higher score in the IDS9-SF reflects more severe internet addiction. The IDS9-SF has been validated among Iranian, Pakistani, and Bangla samples via network analysis [26].

### Data analysis

Participants’ characteristics and basic item properties of the PS-8 were analyzed using descriptive statistics, including means with standard deviations (SDs) and frequencies with percentages. Afterward, three types of psychometric testing (confirmatory factor, network, and Rasch analyses) were used to examine psychometric properties of the PS-8. After verifying the factor structure of the PS-8, internal consistency using Cronbach’s α and McDonald’s ω was examined for the entire PS-8 and potential domains of the PS-8 (if the PS-8 was found to have more than one underlying factor). When Cronbach’s α and McDonald’s ω were higher than 0.7, the internal consistency was deemed satisfactory [36, 37].

### Network analysis

Network analyses can provide insight into items’ structures, positions, and dyadic properties in easy-to-understand patterns [24]. Correlations between PS-8 items may be visualized using lines of different widths to visualize if any two items have strong or weak correlations [25, 26]. With such illustrations, network analyses can provide straightforward information regarding whether an instrument (e.g., the PS-8 in the present study) assesses the same construct in a manner differing from other constructs (e.g., internet addiction). That is, when concurrently using network analysis on two different scales, one can identify if the two different scales assess different concepts.

The network analysis included all 17 items of the PS-8 and IDS9-SF. The required minimum sample size in network analysis based on Leme’s *et al* [38] recommendation was [(17) + (17 × 16/2) = 153]. Using the Extended Bayesian Information Criterion (EBICglasso) as an estimator with 1000 bootstraps, analyses were conducted in Jeffreys’ Amazing Statistics Program (JASP) version 0.15.0.0. In the model, each variable is a node and connections between nodes are edges.

### Confirmatory factor analysis (CFA)

CFA with a diagonally weighted least squares estimator was used to test two potential factor structures of the PS-8: (i) a one-factor structure that has all 8 PS-8 items embedded in the same construct; (ii) a two-factor structure that has the first 4 PS-8 items embedded in the construct of Communication Disturbance and the last 4 PS-8 items in the construct of Phone Obsession.

Several fit indices were used to examine if the proposed factor structures (i.e., one-factor and two-factor structure) had satisfactory data-model fits. A comparative fit index (CFI) and Tucker-Lewis index (TLI) higher than 0.9 together with root mean square error of approximation (RMSEA) and standardized root mean square (SRMR) less than 0.08 indicate satisfactory fit [39–41]. After the factor structures were examined using CFA, the better structure was further tested for measurement invariance across country (i.e., Bangladesh, Iran, and Pakistan) and across males and females. Three nested models were used in the measurement invariance test: (i) configural model that assumes each subgroup having the same factor structure; (ii) metric invariant model (aka weak invariant model) that assumes each subgroup having equivalent factor loadings; and (iii) scalar invariant (aka strong invariant model) that assumes each subgroup having equivalent factor loadings and item intercepts [42, 43]. The equivalence of factor loadings and item intercepts was examined using differences in CFI, RMSEA, and SRMR (i.e., ΔCFI, ΔRMSEA, and ΔSRMR). With ΔCFI > -0.01 together with ΔRMSEA and ΔSRMR < 0.01 [44–46], the equivalence was considered supported and the PS-8 considered as metric or scalar invariant across the testing subgroups. The required minimum sample size in CFA based on the RMSEA (http://quantpsy.org/rmsea/rmsea.htm) was 525 when type I error at 0.05, power at 0.9, null RMSEA at 0, and alternative RMSEA at 0.05. JASP was used for CFA.

### Rasch analysis

Rasch analyses possess a mathematic advantage of converting item scores into continuous scales [27, 28]. With the use of additive unit, Rasch analyses assess if items in an instrument are embedded in a unidimensional concept [29]. Moreover, Rasch analyses can examine if different subgroups interpret item descriptions differently or show differential item functioning (DIF) [30, 31].

If the network analysis and CFA results showed that the PS-8 was unidimensional, all 8 items would be analyzed in the Rasch analysis simultaneously to examine if they all embedded in the same construct. If a two-factor structure of the PS-8 was supported by the network analysis and CFA results, two Rasch models would be constructed: one construct testing the first four items for the Communication Disturbance domain; another testing the last four items for the Phone Obsession domain. Infit and outfit mean square (MnSq) was used to test if each PS-8 item fit in its embedded construct: both infit and outfit MnSq scores ranging between 0.5 and 1.5 indicate good fit [47, 48]. Afterward, DIF of each PS-8 item was assessed to examine if any item had substantial DIF across countries or sex/gender. A substantial DIF was defined as a DIF contrast (i.e., difficulty differences between subgroups) larger than 1 [49]. The required minimum sample size in Rasch analysis based on a five-point Likert scale is between 25 × (5 + 1) = 150 and 100 × (5 + 1) = 600 [50].

## Results

Among the 1902 participants who completed the PS-8, 957 were female (50.4%), 928 were male (48.8%), and 16 did not want to disclose (0.8%). Participants were relatively young (mean age = 26.3 years; SD = 8.1) and relatively equally distributed across the three countries: 534 (28.1%) in Bangladesh, 702 (36.9%) in Iran, and 666 (35.0%) in Pakistan. On average, they spent 6.42 hours (SD = 4.66) using a cellphone per day. Additional participant information is reported in Table 1. The PS-8 item properties are reported in Table 2. In brief, the mean scores of the 8 items were between 2.00 and 3.85; the skewness (-0.91 to 0.86) and kurtosis (-1.24 to -0.15) values were close to normal distribution.

**Table 1:** Descriptive characteristics of the sample (n = 1,902)

Variables		N (100%)/Mean (± SD)
Age, years		26.3 (± 8.1)
Gender		
	Male	928 (48.8%)
	Female	957 (50.4%)
	Prefer not to say	16 (0.8%)
Country		
	Bangladesh	534 (28.1%)
	Iran	702 (36.9%)
	Pakistan	666 (35%)
Marital status		
	Married	533 (28.0%)
	Single	1348 (70.9%)
	Divorced/widowed	21 (1.1%)
Living area	Urban	1499 (78.8%)
	Rural	264 (13.9%)
	Suburb	139 (7.3%)
Current smoker	Yes	297 (15.6%)
Cellphone use per day (in hours)		6.42 (± 4.66)

**Table 2:** Properties of items from the 8-item Phubbing Scale

Item	Mean	SD	Kurtosis	Skewness
1. My eyes start wandering on my phone when I’m together with others	2.26	1.06	-0.54	0.47
2. I am always busy with my mobile phone when I’m with my friends	2.08	1.01	-0.31	0.63
3. People complain about me dealing with my mobile phone	2.00	1.10	-0.15	0.86
4. I’m busy with my mobile phone when I’m with friends	2.36	1.11	-0.57	0.44
5. My phone is always within my reach	3.85	1.24	-0.18	-0.91
6. When I wake up in the morning, I first check the messages on my phone	3.55	1.33	-0.95	-0.48
7. I feel incomplete without my mobile phone	2.85	1.41	-1.25	0.11
8. My mobile phone use increases day by day	2.82	2.85	-1.00	0.18

**Fig. 1:**
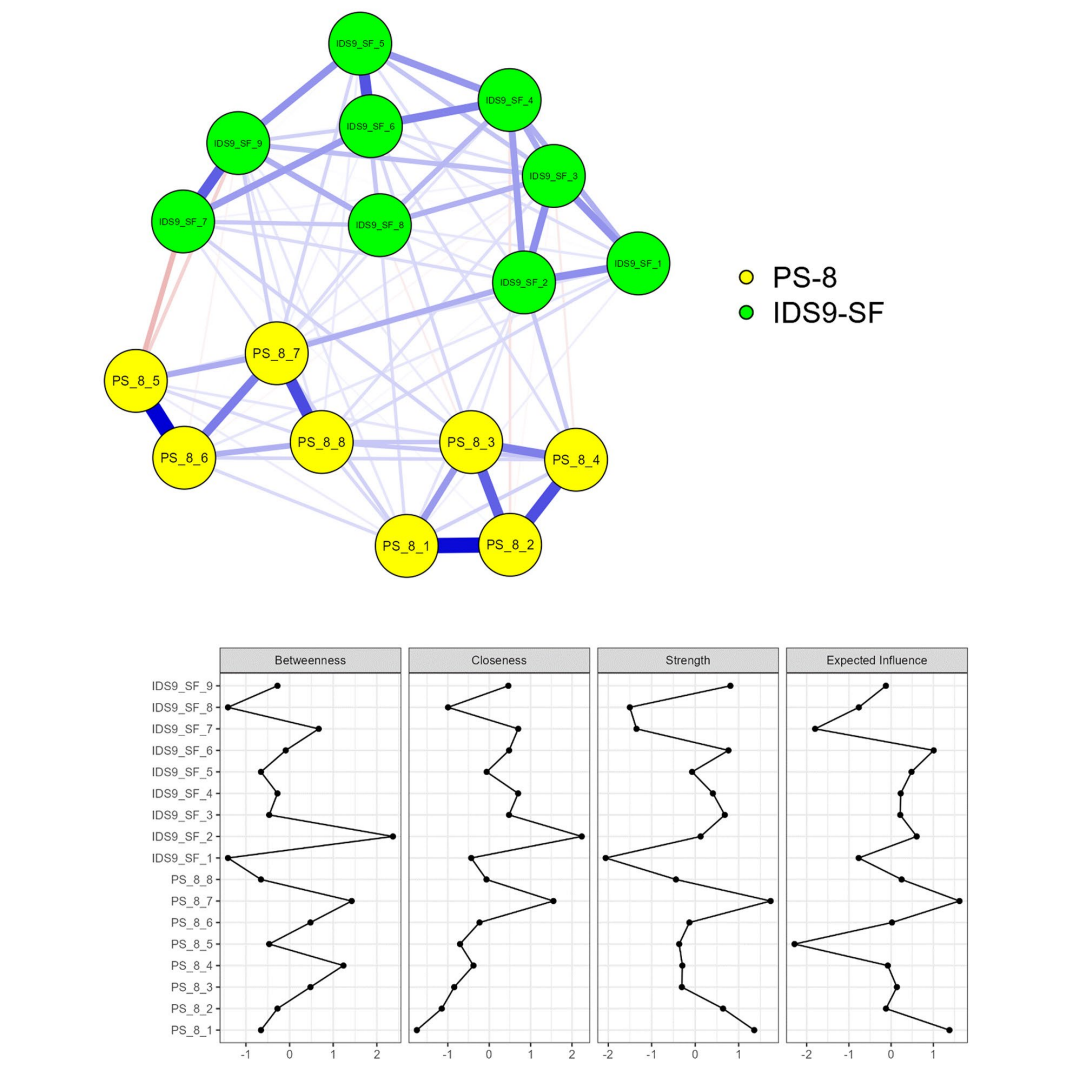
Network of Relationships Between Phubbing and Internet Use Disorder. Note. positive and negative associations are indicated by blue and red lines, respectively

Network analysis showed that all PS-8 items were closely associated, and all IDS9-SF items were closely associated. In addition, two clear factors were identified: one for phubbing and another for internet addiction (Fig. 1). The accuracy and the stability of the estimation in the network analysis model was assessed using bootstrapped the 95% confidence intervals (CIs) of the edge weights. The estimated CIs for most of the edges were narrow, indicating edge-weight accuracy. As shown in Fig. 1 and Supplementary Table S1, there were positive correlations between PS-8 and IDS9-SF items. The nodes of the PS-8 (PS8-1 to 8) and IDS9-SF (IDS1 to 9) items clustered together in different sections of the network. There were noticeable and strong edge connection between IDS2 and PS8-7. Additional results regarding the network analysis can be found in Supplementary materials.

The two-factor structure of the PS-8 was confirmed by CFA (Table 3). Although some fit indices were acceptable for a one-factor structure of the PS-8, some fit indices were unsatisfactory (e.g., RMSEA > 0.08 for each country sample and the entire sample). Unsatisfactory fit indices were not observed for the two-factor structure of the PS-8 (CFI = 0.970 to 0.997; TLI = 0.956 to 0.996; RMSEA = 0.025 to 0.058; and SRMR = 0.038 to 0.076), except for one slightly high value in RMSEA (0.093) among Pakistani participants. Therefore, measurement invariance of the PS-8 was conducted for its two-factor structure. Metric (or weak) invariance was supported for the PS-8 across countries (ΔCFI = 0.014; ΔRMSEA = -0.017; and Δ SRMR = -0.013) and scalar (or strong) invariance was supported across sex/gender (ΔCFI = 0.000 and − 0.001; ΔRMSEA = 0.002 and − 0.003; and Δ SRMR = 0.000 and − 0.003). However, scalar invariance of the PS-8 across countries was not fully supported (ΔCFI = -0.020; ΔRMSEA = 0.019; and Δ SRMR = 0.014) (Table 4).

**Table 3:** Confirmatory factor analysis fit indices and internal consistency for the 8-item Phubbing Scale

	N	Female	Male	Age: Mean (SD)	χ^2^ (df)	CFI	TLI	RMSEA (95%CI)	SRMR	α	ω
**One-factor structure**											
Iran	702	356 (50.7%)	336 (47.9%)	33.25 (8.70)	79.335 (20)	0.979	0.971	0.065 (0.050–0.080)	0.062	0.841	0.836
Bangladesh	534	270 (50.6%)	259 (48.5%)	22.69 (4.58)	153.857 (20)	0.948	0.928	0.112 (0.096–0.129)	0.094	0.847	0.845
Pakistan	666	331 (49.7%)	334 (50.2%)	21.77 (2.50)	369.027 (20)	0.905	0.866	0.162 (0.148–0.177)	0.132	0.856	0.861
All countries	1902	957	928	26.3 (8.1)	524.025 (20)	0.943	0.920	0.115 (0.107–0.141)	0.094	0.846	0.841
**Two-factor structure**											
Iran	702	356 (50.7%)	336 (47.9%)	33.25 (8.70)	27.122 (19)	0.997	0.996	0.025 (0.000-0.044)	0.038	0.843/0.718	0.844/0.724
Bangladesh	534	270 (50.6%)	259 (48.5%)	22.69 (4.58)	37.208 (19)	0.993	0.990	0.042 (0.021–0.062)	0.047	0.845/0.781	0.844/0.792
Pakistan	666	331 (49.7%)	334 (50.2%)	21.77 (2.50)	129.415 (19)	0.970	0.956	0.093 (0.079–0.109)	0.076	0.844/0.848	0.845/0.852
All countries	1902	957	928	26.3 (8.1)	140.240 (19)	0.986	0.980	0.058 (0.049–0.067)	0.049	0.841/0.784	0.841/0.788

df = degree of freedom; CFI = comparative fit index; TLI = Tucker-Lewis index; RMSEA = root mean square residual of approximation; SRMR = standardized root mea square error; α = Cronbach’s α; ω = McDonald’s ω

**Table 4:** Measurement invariance of the 8-item Phubbing Scale in two-factor structure across 3 countries and genders/sexes

	χ^2^	df	CFI	RMSEA	SRMR	ΔCFI	ΔRMSEA	ΔSRMR
**Across Country**								
Configural invariance	385.852	73	0.966	0.082	0.072			
Metric (weak) invariance	252.763	69	0.980	0.065	0.059	0.014	-0.017	-0.013
Scalar (strong) invariance	445.542	81	0.960	0.084	0.073	-0.020	0.019	0.014
**Across gender/sex**								
Configural invariance	191.529	73	0.987	0.051	0.053			
Metric (weak) invariance	189.451	69	0.987	0.053	0.053	0.000	0.002	0.000
Scalar (strong) invariance	208.406	81	0.986	0.050	0.050	-0.001	-0.003	-0.003

df = degree of freedom; CFI = comparative fit index; RMSEA = root mean square error of approximation; SRMR = standardized root mean square residual

The unidimensionality of each domain in the PS-8 was supported by Rasch analysis. For the Communication Disturbance domain, infit MnSq ranged between 0.83 and 1.14, and outfit MnSq ranged between 0.82 and 1.11. For Phone Obsession, infit MnSq ranged between 0.93 and 1.12, and outfit MnSq ranged between 0.90 and 1.17. The absolute DIF contrasts between countries and between sexes/genders were all less than 1, indicating no substantial DIF across countries or sexes/genders (Table 5). Moreover, the internal consistency of the PS-8 (its two factors and the entire PS-8) was satisfactory with both Cronbach’s α and McDonald’s ω higher than 0.7 in each country sample and the entire sample (Table 3).

**Table 5:** Rasch analysis results for the 8-item Phubbing Scale

Item #	Difficulty	Mean square		Differential item functioning			
		Infit	Outfit	I vs. B	I vs. P	B vs. P	M vs. F
Item 1	-0.23	0.94	0.94	0.45	0.62	-0.17	0.06
Item 2	0.25	0.83	0.82	-0.34	-0.27	-0.07	0.12
Item 3	0.47	1.14	1.11	0.54	0.39	0.15	-0.17
Item 4	-0.49	1.08	1.07	-0.60	-0.69	0.10	-0.35
Item 5	-0.89	1.12	1.17	-0.38	-0.51	0.13	-0.03
Item 6	-0.40	0.93	0.90	-0.60	-0.25	-0.35	-0.44
Item 7	0.62	0.93	0.92	0.65	0.76	-0.11	0.07
Item 8	0.67	0.94	1.00	0.24	-0.04	0.28	0.15

I = Iran; B **=** Bangladesh; P = Pakistan; M = male; F = female.

## Discussion

The present study used advanced psychometric testing methods to understand the psychometric properties of the PS-8 across three understudied country populations: Bangladesh, Iran, and Pakistan. Network analysis results showed a clear pattern that phubbing is a different concept from internet addiction. The concept of phubbing assessed via PS-8 could be further classified into two domains. Specifically, network analysis provided visual information and evidence [25, 26] that the eight PS-8 items were different from the nine IDS9-SF items. CFA further supported the two-factor structure of the PS-8 and this two-factor structure was invariant across genders/sexes and countries. Finally, Rasch analysis indicated that the four PS-8 items were embedded in one construct and the last PS-8 items in another. All PS-8 items did not have substantial DIF across gender/sex and country, also indicating that these items are invariant across subgroups. Therefore, the prior proposed two-factor structure for the PS-8 [16, 23] was fully supported by the present study’s findings.

The satisfactory psychometric properties of the PS-8 found in the present study were comparable to those from a recent article assessing the psychometric properties of the Portuguese PS-8 [16]. However, García-Castro et al. [16] only assessed the PS-8 in one Portuguese sample. Although they found the two-factor structure of the PS-8 to be invariant across men and women, their results did not provide evidence regarding whether the PS-8 was invariant across countries. Błachnio et al. [23] examined both the PS and PS-8 regarding measurement invariance across 20 countries. They found that the PS could not satisfy measurement invariance, while the PS-8 could. Although the PS-8 was found to be invariant across countries, Błachnio et al. [23] did not include Iranian and Bangladeshi participants in their sample. Therefore, one cannot conclude if the two-factor structure of PS-8 could be replicated among Iranians and Bangladeshis. The present study thus extended the factor structure evidence of the PS-8 to these groups. More specifically, Błachnio et al. [23] had a Pakistani sample in their study and found that the PS-8 was invariant across Pakistani and other countries’ participants. The present study found the PS-8 to be invariant across Pakistani, Iranian, and Bangladeshi participants; thus, the PS-8 may be invariant across Iran, Bangladesh, and the countries involved in Błachnio et al.’s [23] study. Nevertheless, this possibility is based on indirect evidence, and future studies are needed to test this directly. The present study extended the two-factor structure findings of the PS-8 from CFA using another advanced psychometric testing method (i.e., Rasch analysis). Rasch analyses involve converting ordinal scales into continuous scales [27, 28], and findings supported those from network analysis and CFA. The first four PS-8 items showed appropriate infit and outfit MnSq, indicating that the four items embedded in the same construct. Similarly, the last four PS-8 had appropriate infit and outfit MnSq. Rasch analysis additionally verified measurement invariance findings indicating no substantial DIF items in the PS-8 items.

Study limitations warrant mention. First, the present study did not use any external criterion measures to examine the concurrent validity or criterion-related validity of the PS-8. Therefore, it is unclear if the PS-8 tested among participants from the three countries linked to similar relevant constructs (e.g., smartphone addiction or social media addiction). Second, the present study did not examine test-retest reliability of the PS-8. Therefore, it is unclear if the PS-8 could reproduce similar scores across times when participants would be expected to have no changes in their phubbing behaviors. Third, the present study used online survey to collect data. Therefore, potential participants without access of internet during the study period could not participate. In this regard, the representativeness of the present samples is restricted. In addition, the online survey is a type of self-reports, and is thus subject to social desirability biases.

## Conclusion

In conclusion, the present study suggest that the PS-8 may be a feasible and robust instrument for healthcare providers, especially mental health professionals, to quickly assess and evaluate an individual’s phubbing behaviors. The two-factor structure of the PS-8 was confirmed using different statistical methods, which indicate that its factor structure is stable. Indeed, findings from measurement invariance in CFA and DIF in Rasch analysis all support that the PS-8 is invariant across women and men and the three studied countries (i.e., Bangladesh, Iran, and Pakistan). Therefore, the PS-8 can be used for sex-/gender-related and country comparisons. With the strong psychometric properties of the PS-8, healthcare providers may use it to help identify individuals at risk of having phubbing problems and provide interventions as indicated.

### Electronic Supplementary Material

Below is the link to the electronic supplementary material

Supplementary Material 1: Supplementary Table S1. Edge weights between the 8-item Phubbing Scale (PS-8) and IDS9-SF items among 1902 participants

## Data Availability

The dataset for the study is available from the corresponding author upon reasonable request.
