# Supplementary material for: A phubbing scale tested in Bangladesh, Iran, and Pakistan: confirmatory factor, network, and Rasch analyses
Source: BMC Psychiatry. 2023 Oct 18;23:763. doi: 10.1186/s12888-023-05251-4 (PMC10583412; doi:10.1186/s12888-023-05251-4)

**Supplementary materials**

Supplementary Table S1. Edge weights between the 8-item Phubbing Scale (PS-8) and IDS9-SF items among 1902 participants

| Variable | PS8-1 | PS8-2 | PS8-3 | PS8-4 | PS8-5 | PS8-6 | PS8-7 | PS8-8 | IDS9-1 | IDS9-2 | IDS9-3 | IDS9-4 | IDS9-5 | IDS9-6 | IDS9-7 | IDS9-8 | IDS9-9 |
| --- | --- | --- | --- | --- | --- | --- | --- | --- | --- | --- | --- | --- | --- | --- | --- | --- | --- |
| PS8-1 | 0 |  |  |  |  |  |  |  |  |  |  |  |  |  |  |  |  |
| PS8-2 | 0.405 | 0 |  |  |  |  |  |  |  |  |  |  |  |  |  |  |  |
| PS8-3 | 0.167 | 0.262 | 0 |  |  |  |  |  |  |  |  |  |  |  |  |  |  |
| PS8-4 | 0.074 | 0.0291 | 0.210 | 0 |  |  |  |  |  |  |  |  |  |  |  |  |  |
| PS8-5 | 0.032 | 0 | -0.016 | 0.043 | 0 |  |  |  |  |  |  |  |  |  |  |  |  |
| PS8-6 | 0.062 | 0 | 0 | 0.069 | 0.422 | 0 |  |  |  |  |  |  |  |  |  |  |  |
| PS8-7 | 0.076 | 0 | 0 | 0 | 0.138 | 0.216 | 0 |  |  |  |  |  |  |  |  |  |  |
| PS8-8 | 0.062 | 0 | 0.093 | 0.093 | 0.061 | 0.131 | 0.298 | 0 |  |  |  |  |  |  |  |  |  |
| IDS9-1 | 0.026 | 0 | 0 | 0 | 0.052 | 0.042 | 0 | 0.065 | 0 |  |  |  |  |  |  |  |  |
| IDS9-2 | 0.046 | 0 | 0.042 | 0.092 | 0 | 0 | 0.132 | 0.011 | 0.185 | 0 |  |  |  |  |  |  |  |
| IDS9-3 | 0 | -0.012 | 0 | -0.034 | 0 | -0.014 | 0.034 | 0.055 | 0.179 | 0.185 | 0 |  |  |  |  |  |  |
| IDS9-4 | 0 | -0.044 | 0 | 0 | 0 | 0 | 0.046 | 0 | 0.132 | 0.159 | 0.141 | 0 |  |  |  |  |  |
| IDS9-5 | 0.001 | 0 | 0 | 0 | 0 | 0 | 0.069 | 0.032 | 0 | 0.050 | 0.097 | 0.173 | 0 |  |  |  |  |
| IDS9-6 | 0.031 | 0 | 0.066 | 0.056 | 0 | 0.003 | 0.024 | 0 | 0.056 | 0 | 0.053 | 0.205 | 0.283 | 0 |  |  |  |
| IDS9-7 | 0.046 | 0.014 | 0.072 | 0 | -0.118 | 0 | 0 | 0 | 0 | 0.061 | 0.020 | 0 | 0.007 | 0.164 | 0 |  |  |
| IDS9-8 | 0.058 | -0.028 | 0 | 0 | 0 | 0 | 0.057 | 0 | 0.041 | 0.042 | 0.128 | 0.120 | 0.093 | 0.052 | 0.089 | 0 |  |
| IDS9-9 | 0.042 | 0 | 0.033 | 0 | -0.072 | -0.02 | 0.077 | 0.045 | 0.006 | 0 | 0.110 | 0.014 | 0.179 | 0.077 | 0.265 | 0.134 | 0 |

**Supplementary Figure S1.** Standardized estimates of node clustering in the network among 1902 participants


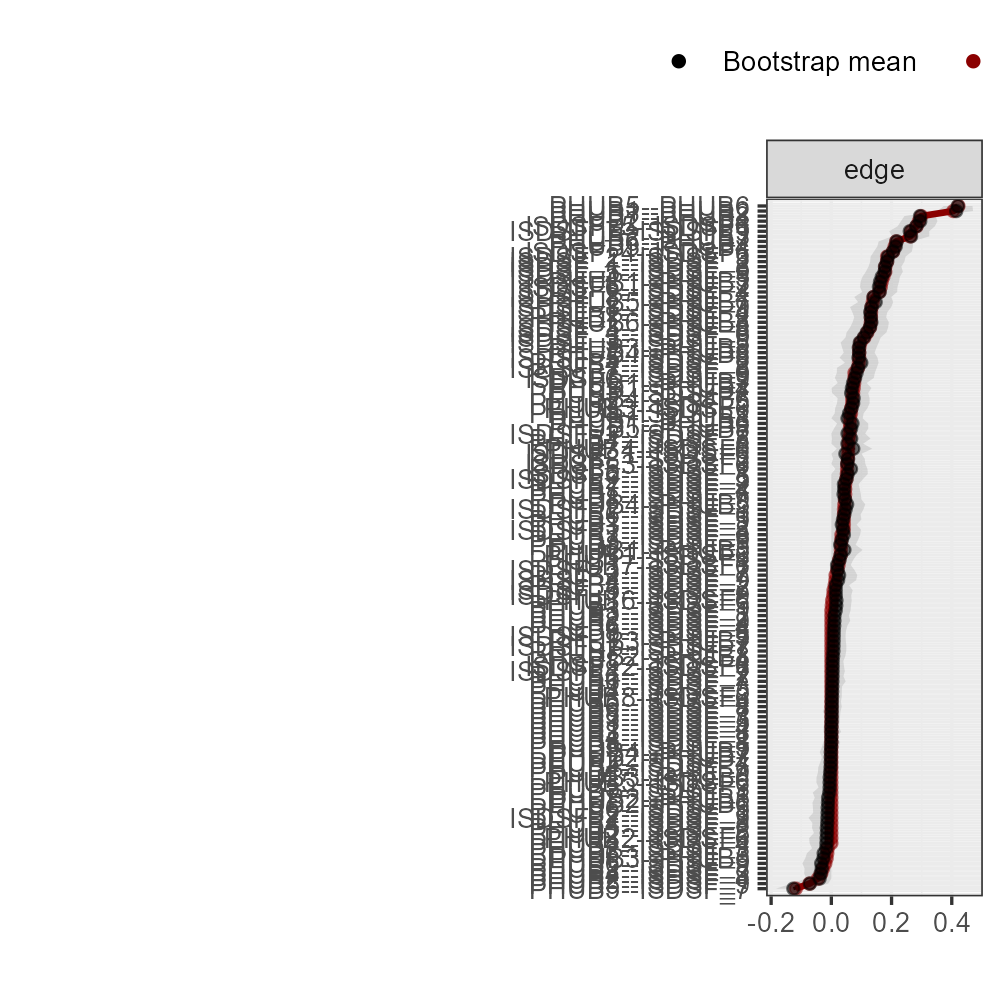

Supplement: Supplementary file 1 — Supplementary Material 1: Supplementary Table S1. Edge weights between the 8-item Phubbing Scale (PS-8) and IDS9-SF items among 1902 participants [file 12888_2023_5251_MOESM1_ESM.doc]
